# Regulation of feeding dynamics by the circadian clock, light and sex in an adult nocturnal insect

**DOI:** 10.3389/fphys.2023.1304626

**Published:** 2024-01-09

**Authors:** Evan Force, Michel B. C. Sokolowski, Caroline Suray, Stéphane Debernard, Abhishek Chatterjee, Matthieu Dacher

**Affiliations:** ^1^ Sorbonne Université, Université Paris-Est Créteil, INRAE, CNRS, IRD, Institute for Ecology and Environmental Sciences of Paris, iEES Paris, Versailles, France; ^2^ Sorbonne Université, Université Paris-Est Créteil, INRAE, CNRS, IRD, Institute for Ecology and Environmental Sciences of Paris, iEES Paris, Paris, France; ^3^ Université de Picardie—Jules Verne, Amiens, France

**Keywords:** *Agrotis ipsilon*, Lepidoptera, feeding behavior, circadian behavior, developmental timing, automated feeding cage

## Abstract

Animals invest crucial resources in foraging to support development, sustenance, and reproduction. Foraging and feeding behaviors are rhythmically expressed by most insects. Rhythmic behaviors are modified by exogenous factors like temperature and photoperiod, and internal factors such as the physiological status of the individual. However, the interactions between these factors and the circadian clock to pattern feeding behavior remains elusive. As *Drosophila*, a standard insect model, spends nearly all its life on food, we rather chose to focus on the adults of a non-model insect, *Agrotis ipsilon*, a nocturnal cosmopolitan crop pest moth having structured feeding activity. Our study aimed to explore the impact of environmental cues on directly measured feeding behavior rhythms. We took advantage of a new experimental set-up, mimicking an artificial flower, allowing us to specifically monitor feeding behavior in a naturalistic setting, *e.g.*, the need to enter a flower to get food. We show that the frequency of flower visits is under the control of the circadian clock in males and females. Feeding behavior occurs only during the scotophase, informed by internal clock status and external photic input, and females start to visit flowers earlier than males. Shorter duration visits predominate as the night progresses. Importantly, food availability reorganizes the microstructure of feeding behavior, revealing its plasticity. Interestingly, males show a constant number of daily visits during the 5 days of adult life whereas females decrease visitations after the third day of adult life. Taken together, our results provide evidence that the rhythmicity of feeding behavior is sexually dimorphic and controlled by photoperiodic conditions through circadian clock-dependent and independent pathways. In addition, the use of the new experimental set-up provides future opportunities to examine the regulatory mechanisms of feeding behavior paving the way to investigate complex relationships between feeding, mating, and sleep-wake rhythms in insects.

## 1 Introduction

Earth rotation leads to a 24-h periodicity in the abiotic environment. During evolution, nearly every organism acquired circadian clocks to adapt to this predictable periodic change (Eelderink-Chen et al., 2021; for a review, see Paranjpe and Sharma, 2005). The circadian clock is a predictive internal model that anticipates environmental changes, allowing the organism to cope with these changes on a daily basis. The phase and period of this internal clock are synchronized to cycling environmental cues such as light, tide, or temperature, which provide an external time reference for tuning the internal clock. In insects, the clock drives overt circadian rhythms in innate and learned behaviors including sleep and arousal, courtship and mating, foraging, oviposition, and memory formation, while also sustaining seasonally manifested behaviors like migration and diapause (for reviews, see Saunders, 2002; Lazzari and Insausti, 2008; Tomioka et al., 2012; Brady et al., 2021). Furthermore, the circadian clock of insects also regulates developmental transitions like metamorphosis and hatching (Sauman et al., 1996; Vafopoulou and Steel, 2012; Mark et al., 2021; Matsuda, 2023; for reviews, see Saunders, 2002; Lazzari and Insausti, 2008; Allada and Chung, 2010; Tomioka et al., 2012; Franco et al., 2018; Brady et al., 2021).

Studies of the clock in *Drosophila* led to the discovery of shared underlying molecular mechanisms (for a review, see De and Chatterjee, 2022), and non-model insects enrich our understanding of its evolution and adaptation to ecological constraints (for reviews, see Sandrelli et al., 2008; Denlinger et al., 2017). Moths, which belong to the order Lepidoptera, constitute 10% of all living species; their nocturnal behavioral activity distinguishes them from the diurnal *Drosophila*, allowing scientists to gain comparative insight (Niepoth et al., 2018; for a review, see Groot, 2014). Different moth species such as *Manduca sexta*, *Hyalophora cecropia*, *Antheraea pernyi*, and *Spodoptera litura* show nearly 24 h rhythms in locomotor activity that peaks at night in light-dark (LD) conditions, at the subjective night in dark-dark (DD) conditions, and becomes arrhythmic in light-light (LL) conditions (Truman, 1974; Broadhead et al., 2017; Zhang et al., 2021). Besides locomotor activity, the larvae of noctuid moths *Spodoptera littoralis*, *Spodoptera exigua*, and *Spodoptera littura* also exhibit daily rhythms in food consumption mostly in LD (Kim and Hong, 2015; Suszczynska et al., 2017; Zhang et al., 2021), while their feeding rhythms rapidly dampen in DD conditions. However, owing to a lack of suitable assays, it remains hitherto unknown whether the circadian clock drives a bona-fide feeding rhythm in adult moths. This is a relevant point, as they occupy a completely different trophic niche (nectar drinking in adults vs plant eating in larvae) and uses food-derived energy in distinct physiological functions compared to the larva: development for larva and reproduction for adults.

A seminal study in *Drosophila* showed that adult feeding follows a 24-h circadian oscillation with a peak in the morning, regulated by clocks in the fat body, which is an insect analog of the liver, adipose tissue, and pancreas (Xu et al., 2008). By knocking out the clockwork in digestive/metabolic tissues Xu *et al.* (2008) also showed that feeding and global locomotor rhythms can be decoupled. However, within 24 h, the daily time of food intake is not clearly distinguished. Indeed, subsequent studies in the LD condition showed varying degrees of bimodality in feeding habits coinciding with the flies’ morning and evening locomotor activity peaks (Seay and Thummel, 2011; Ro et al., 2014; Dreyer et al., 2019; Niu et al., 2021). Moreover, tracer methods (Xu et al., 2008; Seay and Thummel, 2011), and electromagnetic methods (Ro et al., 2014; Dreyer et al., 2019) neither directly estimate feeding behavior nor use ethologically relevant naturalistic settings. As a result, general mobility and feeding are not properly disentangled.

Adult moths offer unique opportunities to study feeding behavior and its rhythm given their nocturnal ecology, their interspecific diversity from polyphagy to host preference (Cheng et al., 2017; Coates et al., 2023; for a review, see Jaenike, 1990) and for some of them their long-distance seasonal migration between geographically dissimilar places (Causse et al., 1989; Alarcón et al., 2008). Moreover, while *Drosophila* spends all their life on or near rotting fruits from embryonic development to death, moths forage by traveling up to several kilometers per night to drink nectar on the go (Showers et al., 1989). This distinct behavioral ecology makes the feeding rhythms of adult moths an interesting node for comparative purpose. However, this also necessitates the development of precise, automated, direct assays for quantifying long-term feeding dynamics with high temporal resolution, in ethologically pertinent behavioral chambers. We took up this challenge with adult *Agrotis ipsilon*, a cosmopolitan crop pest moth of the Noctuidae family. To do so, we developed a new experimental set-up to monitor feeding behavior while controlling other parameters (light conditions, food availability, sex of the animals) over several days. Importantly, we also recorded general mobility using a locomotor activity monitoring system, to differentiate feeding-related activities from global locomotor activity.

## 2 Materials and methods

### 2.1 Insects

**FIGURE 1:**
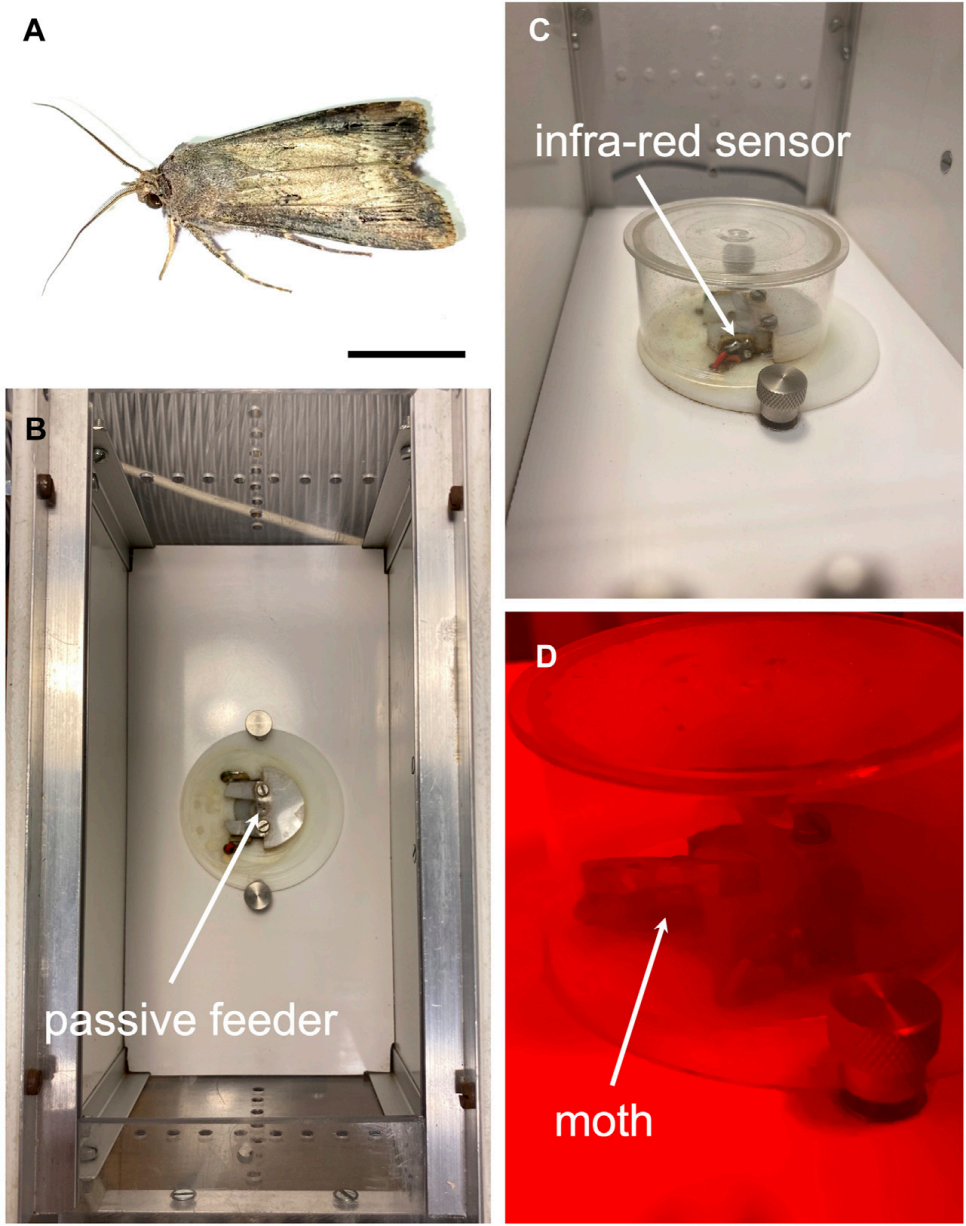
Photographs of the new device for automatic measurement of feeding behavior parameters of adult Lepidoptera, the moth-box. **(A)** Adult male *Agrotis ipsilon*, scale bar = 1 cm. **(B)** Open experimental device mimicking an artificial flower in the above view, including a sucrose dispenser and an infrared motion sensor detecting moth entrance in the mimetic short flower corolla. **(C)** Closed experimental device in side view. **(D)** Artificial flower visited by a moth during the scotophase (photo taken under red light). The infrared sensor detects the entry of the moth at its thorax.

Our work was carried out on *A. ipsilon* (Noctuidae, Lepidoptera) (Figure 1A) after their emergence. This insect is indigenous to the south of France. They were bred in our laboratory at INRAE in Versailles, France. Rearing was carried out according to the protocol described by Force *et al.* (2023); males and females were separated from pupation so that males are completely naïve regarding female sex-pheromone. Every Monday morning, among the emerged male or female moths, we collected and placed 1 individual in each of the five automated feeding cages (the individuals were all of the same sex during an experiment). The collection day was considered the first day of post-emergence life (noted D1). Experiments were conducted during the first 5 days of adult life (*i.e*., from D1 to D5), without any mortality since fasting moths survive at least 7 days. Furthermore, upon emergence, moths readily detect (by their antennae and legs) sugar solutions and feed on them (Hostachy et al., 2019); and it is during the first days of adult life that the sexual maturation of these insects takes place. Indeed, with a sucrose diet, females are sexually mature from the third day while males are sexually mature on the fifth post-emergence day (Gemeno and Haynes, 2000; Force et al., 2023).

### 2.2 Moth-box: an automated feeding cage

Feeding behavior monitoring of *A. ipsilon* was carried out using the moth-box, an automated feeding cage (Figures 1B–D), similar to the bee-box previously developed (Riva et al., 2018). It is a 20 × 10 × 9.5 cm box made of PVC plates for the walls and an opaque white polycarbonate plate for the floor. The plates were glued with transparent polystyrene. Ventilation was ensured by 32 holes of 3.5 mm in diameter on the walls. Each of the cages was equipped with a passive feeder enclosed in a transparent plastic circular box 5 cm in diameter and 2.5 cm in height, large enough to avoid constraining insect locomotion (wing deployment and on-site flight possible) (Figures 1C, D). A passive feeder rather than a dosing pump has been used to prevent the sugar solution to stick and plug the dispensing nozzle, which is highly probable with the very low consumption of only one insect per cage. As a result, this device did not allow food consumption to be quantified; rather, entrance in the feeder was used as a proxy of food consumption. The passive feeder consisted of a 30 mm diameter polyamide disc pierced in its center with an orifice (6 mm in diameter and 12 mm long), and a beveled tunnel 10 mm wide at the outside and 6 mm wide in the center. It was covered with a transparent plastic sheet. In addition, in the center of the tunnel, an infrared motion sensor was set and triggered as soon as the moth entered the artificial flower (Figure 1D). Most nectarivorous Lepidoptera are opportunistic flower visitors and probe flowers of different sizes and shapes (Krenn, 2010). *A. ipsilon* has a short proboscis, the length of which is approximately half the total length of the insect. The morphology of its proboscis seems adapted to feeding from short-corolla flowers as suggested by studies in other moths (Krenn, 1990; 2010). Thus, the shape of the passive feeder imitating a flower with a short corolla seems well suited to accessing food in the center of the device, probably encouraging *A. ispilon* to enter the artificial flower. Unless otherwise mentioned, moths had *ad libitum* access to a 12% sucrose solution (mass/mass or 13.6% mass/volume) (Sigma-Aldrich, Saint-Quentin-Fallavier, France), similar to the nectar foraged in a natural setting (Tiedge and Lohaus, 2017; for reviews, see Roy et al., 2017; Nicolson, 2022).

We had five automated cages operating in parallel, which were in a chamber at controlled conditions (23°C, 50%–60% relative humidity). Each of the cages was occupied by a single individual. The cages were used for feeding behavior monitoring, by measuring specifically the number of visits as a function of time as well as the duration of each visit, with the infrared sensor inside the artificial flower; while the actual consumption is not directly measured, we assumed these parameters are correlated to it. These experiments were performed on moths during the first 5 days of adult life (D1 to D5).

Data were recorded as text on a micro-SD card. The management of the experimental process was carried out automatically by a microcontroller (P8X32A) equipped with a clock making it possible to annotate each behavioral event with a date/time. At the end of an experimental session, data were sent to a computer with a USB wire using Termites RS232 terminal software.

### 2.3 Locomotor activity monitor system

Besides the moth-box, in some experiments, we also used a Locomotor Activity Monitor (Model LAM25). This basic system consisted of a data collection computer, power interface unit PSIU9, activity monitors, tubes with caps, and an incubator (23°C, 50%–60% relative humidity). LAM25 and Power Interface Unit PSIU9 come from Trikinetics Inc. (Waltham, MA, USA). LAM25 monitoring unit has 32 independent channels (25 mm in diameter), into which polymeric methyl methacrylate (PMMA) sample tubes with an inner diameter of 21 mm and a length of 100 mm fit. The ends of these tubes were obstructed by cotton wool soaked in nutrient solution (sucrose 12%, as in the moth-box) unless otherwise mentioned. Locomotor activity of the moth in the tube is measured using infrared beams and sensors. LAM25 monitors were connected to the computer with the PSIU9 power interface unit. The DAMSystem3 (Waltham, MA, United States) data collection program was configured to collect 1-min bin data from each activity monitor and saved the data on a hard drive.

### 2.4 Experiments

The moth-box specifically measures the number and duration of visits to the artificial flower. A first experiment was carried out in males to evaluate the effect of the presence or absence of sugar solution inside the artificial flower on these parameters, under 16 h light and 8 h darkness (16L:8D condition); the scotophase started at 10 a.m. Sugar solution was available either from D1 to D5 (first cohort) or only on D1 and D2 (second cohort). Thus, we will have measures of flower entrance frequency and duration with and without food, so as to confirm these parameters are correlated to the presence of food. In addition, to rule out an effect of starvation on locomotion when animals were deprived of food, general mobility was assessed using other male moths set in the LAM25, in the same 16L:8D pattern and the same sugar solution availability.

A second experiment using the moth-box consisted in measuring the above-described parameters in female moths subjected to a 16L:8D photoperiod and whose sugar solution was available from D1 to D5. They were compared to the corresponding male group from the previous experiment.

Finally, a third experiment was performed with the moth-box in male moths to assess the effects of a variation in photoperiod on the previous behavioral feeding parameters. Animals had *ad libitum* access to the sugar solution. Four conditions were tested.

• 24L:0D from D1 to D5 (noted LL);
• 0L:24D from D1 to D5 (noted DD);
• 16L:8D on D1, then 24L:0D from D2 to D4 and again 16L:8D on D5 (noted LD-LL);
• 16L:8D on D1, then 0L:24D from D2 to D4 and again 16L:8D on D5 (noted LD-DD).

These groups allow us to evaluate the impact of photoperiod on the number and duration of artificial flower visits.

### 2.5 Data processing and analysis

Statistical data analysis was performed using R 4.1.2 (R Core Team, 2021) with RStudio 2021.09.2. The sample sizes were between 5 and 14 individuals, and are reported in the legend of each figure. The alpha risk was 0.05.

Analysis of the day-to-day visit number within a same cohort was performed by a Friedman’s tests followed by a Nemenyi’s *post hoc* tests; comparisons between two cohorts was performed using Wilcoxon’s sum of rank tests.

Fisher-Snedecor’s test was used to compare the variance of visit durations inside the artificial flower. In addition, we carried out analysis of the distributions of visit durations by time slot of 3 h over the entire scotophase by using a Poison mixed-model. These three-time slots were the beginning (10 h–12 h), middle (13 h–15 h), and end (16 h–18 h) of the scotophase. The durations were binned as follows: equal to 1 s, between 2 and 20 s, between 21 and 70 s, and finally more than 71 s up to 200 s (duration from which we considered that the moth was in a state of rest) (Supplementary Figure S1).

Cosinor (Refinetti et al., 2007) and ARSER (Wu et al., 2016) models were used out to determine a circadian rhythm about the daily visits of artificial flower per cohort. Once a rhythm has been significantly determined, we used periodic modeling (encompassing a potential dampening along the experiment, *i.e*., a progressive disappearance of the period) to estimate the phase and period of the rhythm. To compare the different conditions, we used a nonlinear model (nlme package in R):

$$\text{Visit}=\left(A*\exp\left(\gamma t\right)+B\right)*\left(1+\cos\left(2\pi \frac{\;t-\phi \;}{\tau }\right)\right)+\left(at+b\right)$$

where *t* is the time (in hours), γ is the damping parameter, τ is the period (which should be around 24 h for circadian rhythm), ϕ is the phase (time of maximum daily consumption) and A, B, a, and b are used to adjust the equation and compensate for the baseline (Zielinski et al., 2014; De Los Santos et al., 2020). B, a, and b were random factors used to account for repeated measurements. The parameters A, γ, τ, and ϕ were compared between the different groups; first, they were tested against 0 in a reference group, and then potential differences from this reference group were tested for other groups.

### 2.6 Ethics note

French legislation for animal welfare is based on that of the European Union (directive 2010/63/EU) according to which no invertebrate other than cephalopods benefit from ethical protection. However, all the experiments were carried out with care, limiting stressful situations for the animals as much as possible.

## 3 Results

### 3.1 Feeding and locomotor rhythms can be dissociated using the moth-box by analyzing long-term feeding dynamics

**FIGURE 2:**
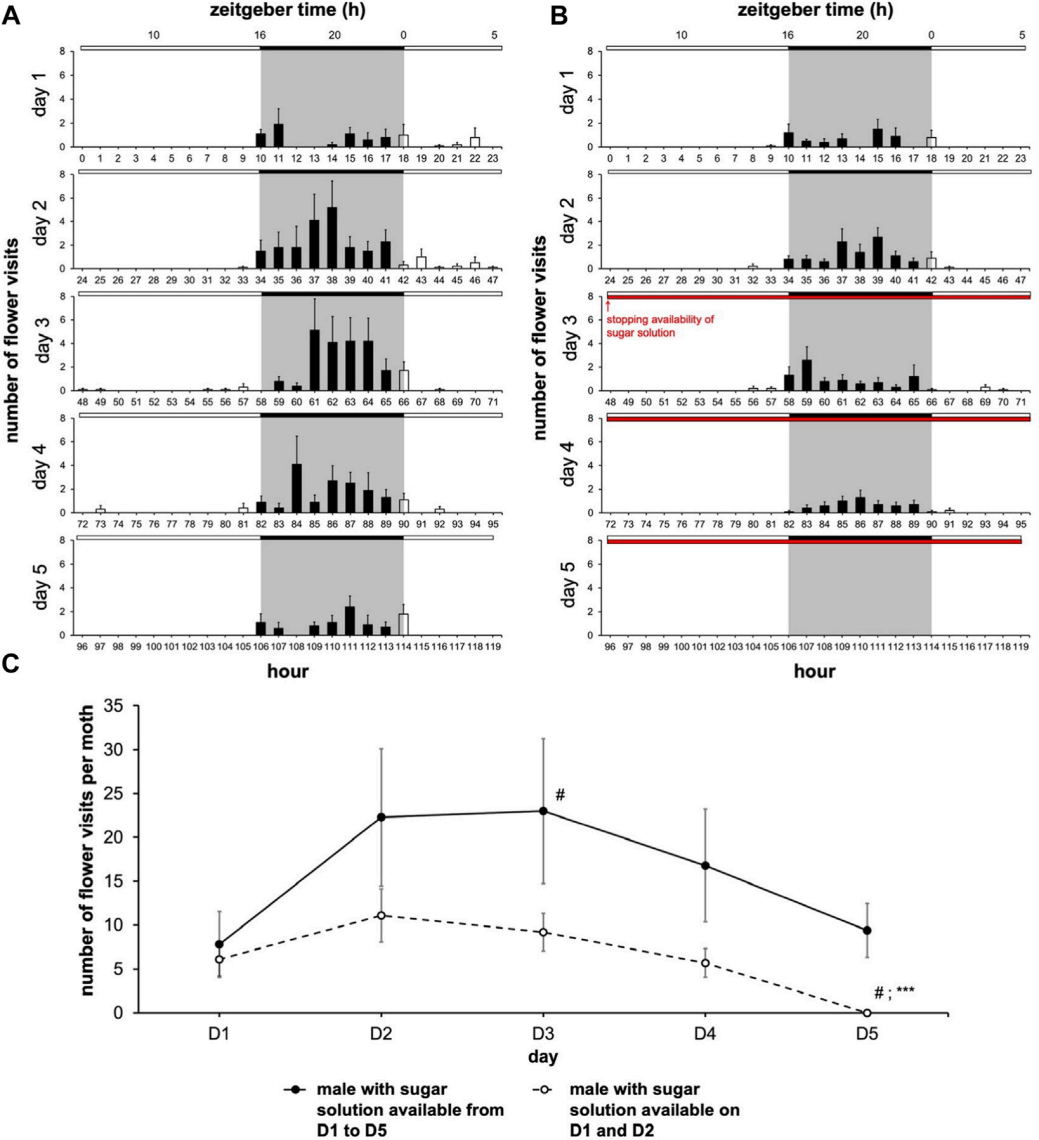
Male moth’ feeding rhythm depends on the availability of sugar solution in artificial flower. The experiment was carried out over 5 days with a photoperiod 16L:8D (*n* = 9 for water cohort; *n* = 10 for sodium cohort). **(A,B)** Diagrams for males with a sugar solution available during the 5 days of experimentation **(A)**, and for males with a sugar solution available during the first 2 days of experimentation **(B)** (full bar, flower visit during scotophase; empty bar, flower visit during photophase; red horizontal bar, stopping the delivery of sugar solution). **(C)** Mean visit number per moth for each of the two cohorts depending on the days (solid line, male with a sugar solution available from D1 to D5; dashed line, male with a sugar solution available on D1 and D2). Values correspond to the mean ± SEM. A Nemenyi’s test was performed to compare the visit number within a same cohort (#, *p < 0.05*). A Wilcoxon’s test (***, *p < 0.001*) was performed to compare the visit number between the two cohorts; statistical data are presented in Supplementary Table S1.

**FIGURE 3:**
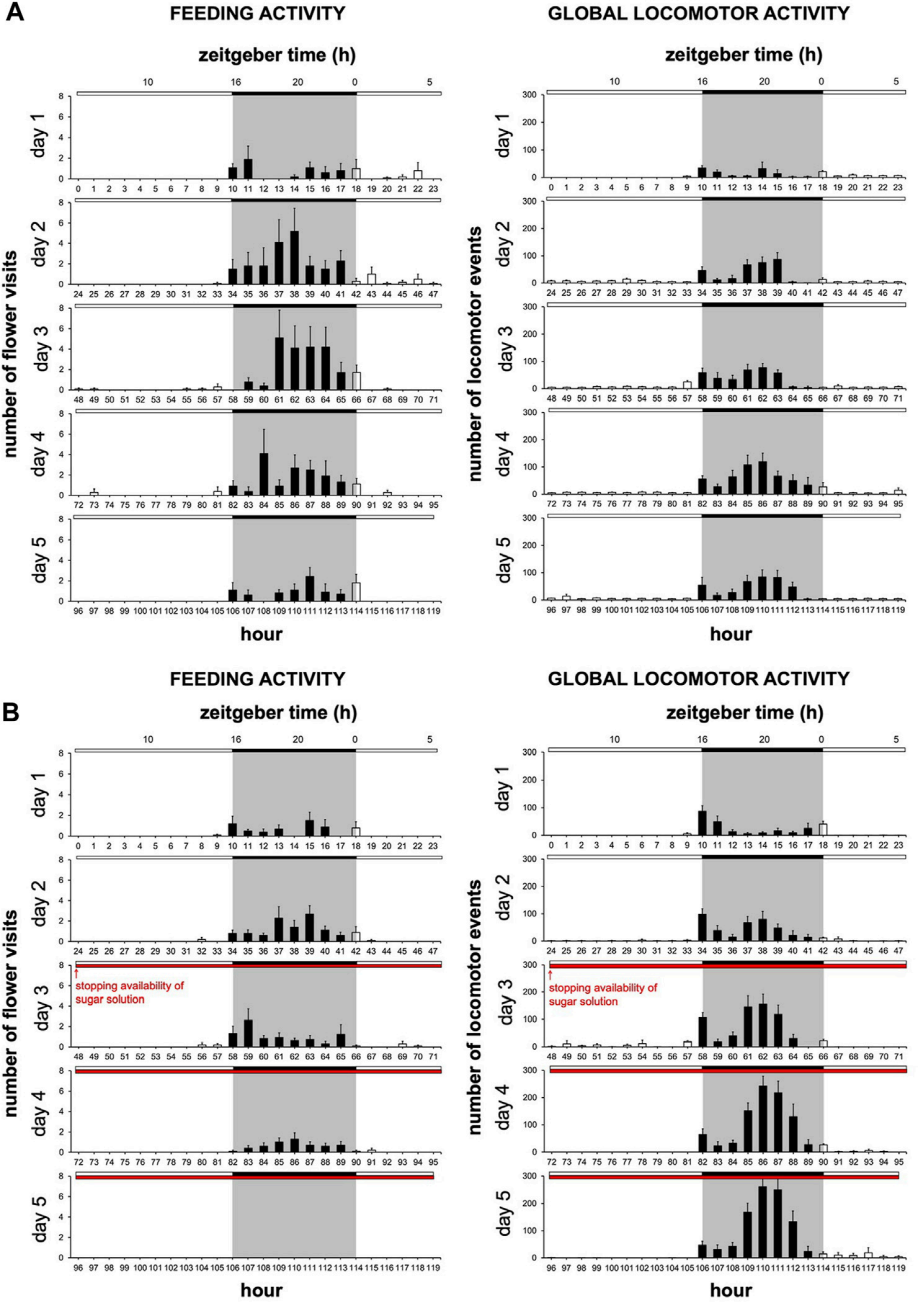
Feeding and locomotor rhythms can be dissociated from each other as soon as food distribution stops. Diagrams for feeding activity assessed in the moth-box (*n* = 10 for each cohort) and global locomotor activity assessed in the LAM25 (*n* = 14 for each cohort) in males with a sugar solution available from D1 to D5 **(A)**, and in males with a sugar solution available on D1 and D2 **(B)** (full bar, event during scotophase; empty bar, event during photophase; red horizontal bar, stopping the delivery of sugar solution). Values correspond to the mean ± SEM. Diagrams for feeding activity are the same as in Figures 2A, B.

We set out to determine whether the moth-box system that we developed provides a specific readout of feeding behavior or merely reflects the spontaneous global locomotor activity of the insect. To resolve this, we assessed the effect of the absence of food within the artificial flower of the moth-box. We reasoned that feeding dynamics should be strongly affected by the absence of food while the global locomotor activity rhythms in moths may not acutely depend on food availability in the short term. Moths with a sugar solution available during the 5 days of monitoring (Figure 2A) showed no significant variation in the frequency of flower visits except between the first and the third days (Nemenyi’s test, *p = 0.020*) (Figure 2C). On the other hand, moths with a sugar solution available only during the first 2 days (Figure 2B) performed a reduced number of visits seeming to start from the third day until a full cessation: number visits on the fifth day were different from all the other days (Nemenyi’s test, *p ≤ 0.025*) (Figure 2C). Moreover, on the fifth day, moths with a sugar solution available *ad libitum* visited the artificial flower significantly more compared to the males with a sugar solution only available on D1 and D2 (Wilcoxon’s test, W = 100.00, *p < 0.0001*) (Figure 2C; Supplementary Table S1). In the two cohorts, neither mortality nor global locomotor activity (assessed in the LAM25) depended on the feeding status (Figure 3).

**FIGURE 4:**
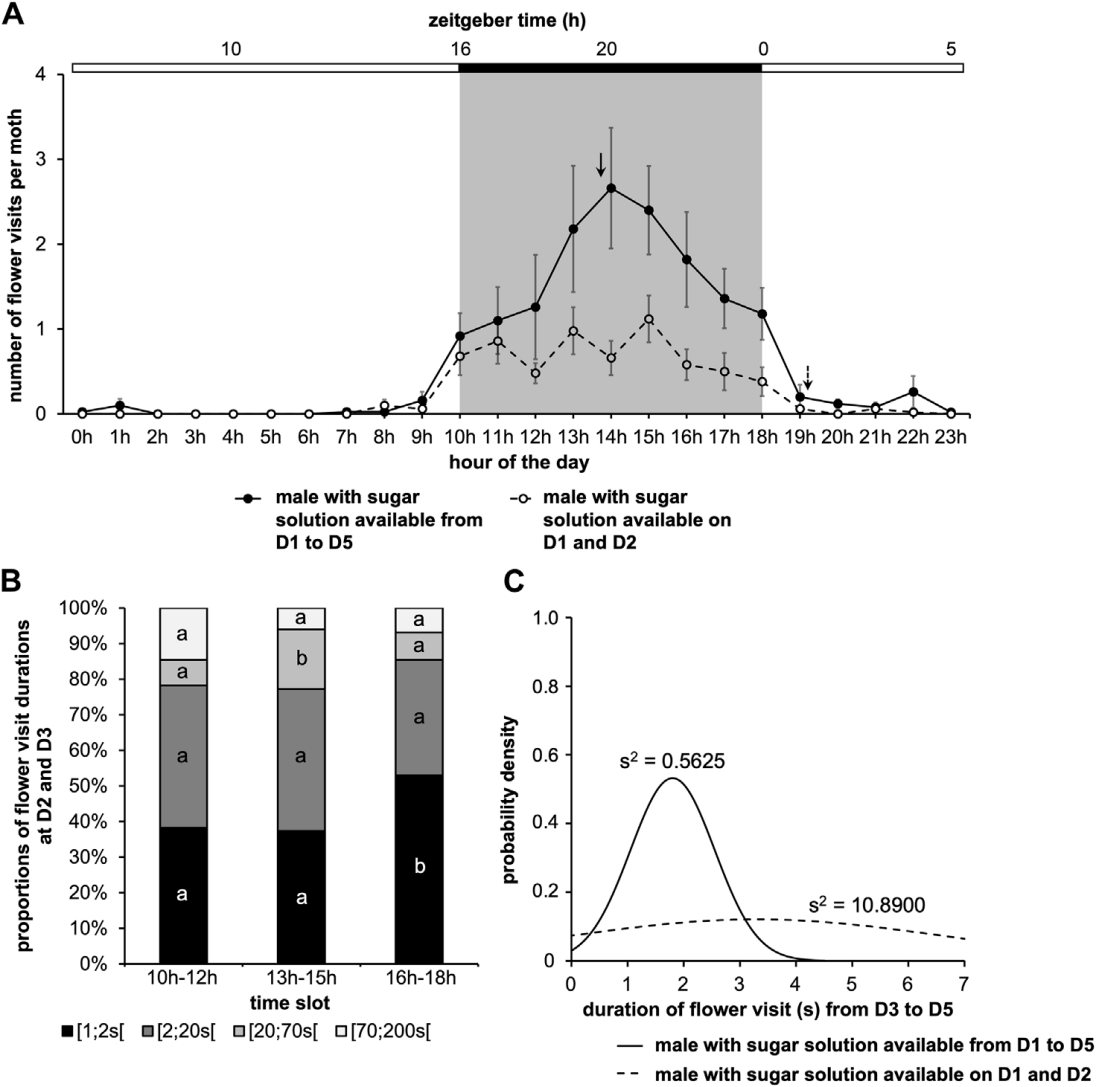
Duration of flower visit is regulated by the circadian clock and sugar solution availability. The experiment was carried out over 5 days with a photoperiod 16L:8D. A first cohort was with a sugar solution available during the 5 days of experimentation; a second cohort was with a sugar solution available during the first 2 days of experimentation (same data as in Figure 2). **(A)** Mean visit number per moth for each of the two cohorts according to the time of day, the 5 days of experimentation are pooled (solid line, male with a sugar solution available from D1 to D5; dashed line, male with a sugar solution available on D1 and D2; solid arrow, phase for males with a sugar solution available from D1 to D5; dashed arrow, phase for males with a sugar solution available on D1 and D2). A statistical analysis of the circadian rhythm was made to compare the two cohorts, statistical data are presented in Supplementary Table S2. Values correspond to the mean ± SEM (*n* = 10 for each cohort). **(B)** Distribution of flower visit durations (data from D2 and D3 are cumulated) for males with a sugar solution available from D1 to D5. Letters indicate the statistical differences after a Poisson mixed-model. **(C)** Duration of flower visit from D3 to D5 according to the two cohorts (solid line, male with a sugar solution available from D1 to D5; dashed line, male with a sugar solution available on D1 and D2). Curves represent normal laws fitted to the data (s^2^ is the variance). A Fisher-Snedecor’s test was performed to analyze the variance of data.

Analysis of the rhythm of feeding behavior in males during the 5 days of experimentation showed scotophase-restricted feeding. For males with a sugar solution available during from D1 to D5, the rhythms of feeding behavior (Cosinor & ARSER models, *p < 0.0001*) exhibited a peak phase between 1 and 3 p.m., *i.e.*, ZT19-21 (*p < 0.0001*), in the middle of the 8-h long scotophase, whereas for males with a sugar solution available on D1 and D2, the peak phase is around 7 p.m., *i.e.*, ZT1 (*p < 0.0001*). Moreover, the periods were also different (*p < 0.0001*) (Figure 4A; Supplementary Table S2).

We then asked whether the distribution of flower visits binned by duration varied over the course of a day. We observed that in the middle of the night, moths had significantly longer (20–70 s duration) and more frequent visits, while toward the end of the night, the prevalence of shorter 1s long flower visits increased (Poisson mixed-model, 6 degrees of freedom χ^2^ = 16.40, *p = 0.012*) (Figure 4B). Overall, the daily changes in feeding frequency (Figure 4A) were, however, more apparent than the changes in flower visit duration (Figure 4B). We also assessed the visit duration over the last 3 days of the study for males with a sugar solution available *ad libitum* and males with a sugar solution available on D1 and D2 (Figure 4C). Here also, the absence of sugar solution in the artificial flower did change the flower visit duration by making it more variable (Fisher-Snedecor’s test, F_9,9_ = 0.05, *p = 0.0001*).

### 3.2 Feeding behavior is sexually dimorphic

**FIGURE 5:**
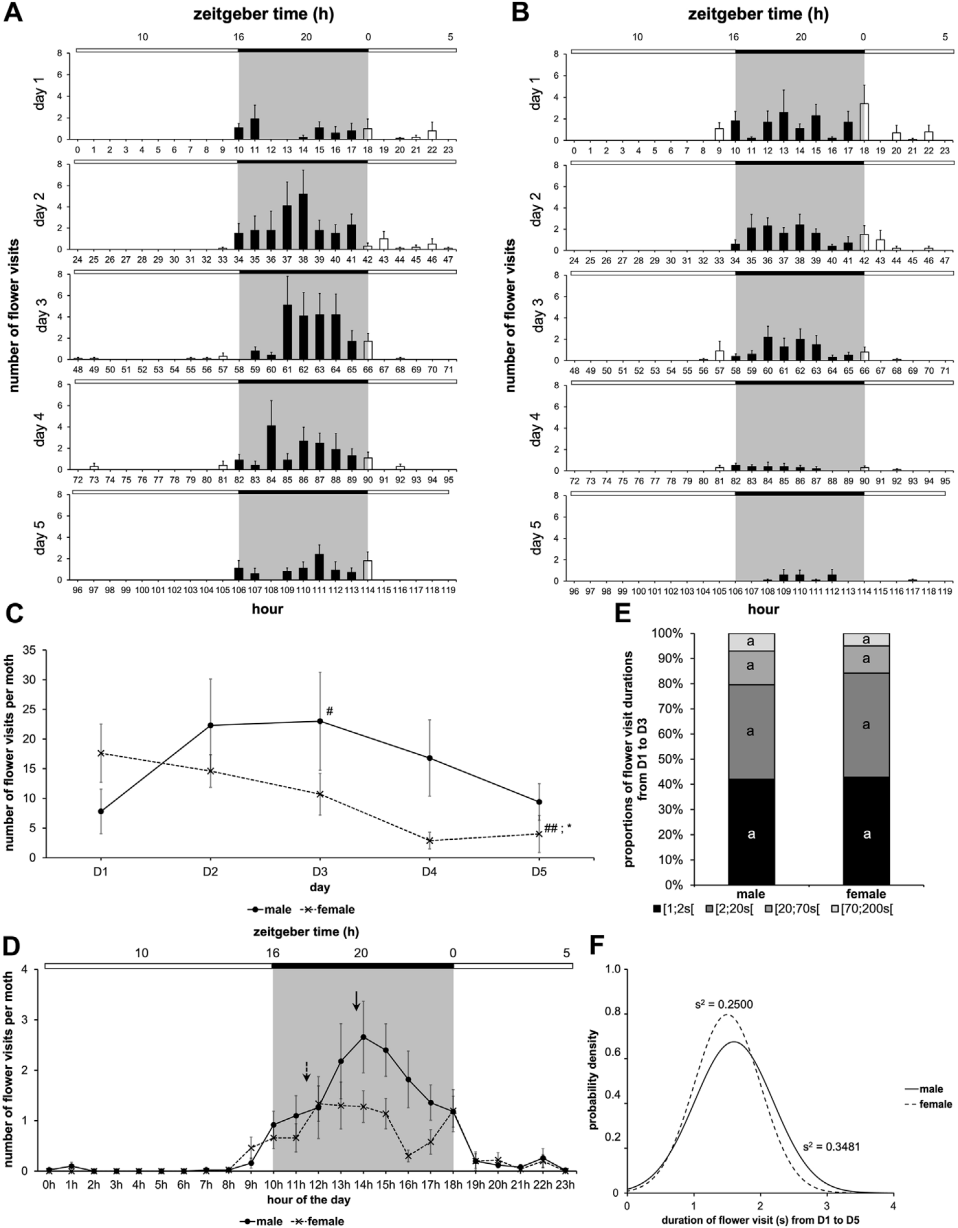
Feeding behavior according to the sex of moths. Data from males in Figure 2A are compared to results from a cohort of female treated the same way. Values correspond to the mean ± SEM (*n* = 10 for each cohort, details as in Figure 2A). Diagram plots results for male **(A)** and female **(B)** with a sugar solution available during the 5 days of experimentation (full bar, flower visit during scotophase; empty bar, flower visit during photophase). **(C)** Mean visit number per moth for each of the two cohorts depending on the days (solid line, male; dashed line, female). A Nemenyi’s test was performed to compare the visit number within a same cohort (#, *p < 0.05*; ##, *p < 0.01*). A Wilcoxon’s test (*, *p < 0.05*) was performed to compare the two cohorts, and statistical data are presented in Supplementary Table S3. **(D)** Mean visit number per moth for each of the two cohorts according to the time of day, the 5 days of experimentation are cumulated (solid line, male; dashed line, female; solid arrow, phase for male; dashed arrow, phase for female). A statistical analysis of the circadian rhythm was made to compare the two cohorts, and statistical data are presented in Supplementary Table S4. **(E)** Proportions of food intake durations (all data from D1 to D5 are cumulated) for males and females. Letters indicate the statistical differences after a Poisson mixed-model. **(F)** Duration of flower visit from D1 to D5 according to the two cohorts (solid line, male; dashed line, female). Curves represent normal laws fitted to the data (s^2^ is the variance). A Fisher-Snedecor’s test was performed to analyze the variance of data.

We then examined the dynamics of feeding in adult males and females of *A. ipsilon* by comparing previous data (Figure 2A) to the result obtained by a cohort of female. For females, flower visit frequency was high for the first 3 days, then it started to decrease and became significantly lower on the fifth day (Nemenyi’s test, *p ≤ 0.007*) (Figure 5A–C). A comparison of the two cohorts also indicates that on the fifth day, females visited the artificial flower less often than males (Wilcoxon’s test, W = 83.00, *p = 0.012*) (Figure 5C; Supplementary Table S3). In addition, in the two cohorts, we observed no mortality over the 5 days of the study (data not shown).

The study of the feeding rhythm of males and females showed flower visits restricted to the scotophase (Cosinor & ARSER models, *p < 0.0001*) (Figures 5A, B). Moreover, for the two cohorts, as expected, the period in LD cycles was 24 h (*p < 0.0001*). On the other hand, the phase was between 1 p.m. and 2 p.m., *i.e.*, ZT19-20 for males (*p < 0.0001*), and around 11 a.m., *i.e.*, ZT17 for females (*p < 0.0001*) (Figure 5D; Supplementary Table S4). Therefore, during the scotophase, females search for food before males.

Then, we compared the different proportions of flower visit duration during the first 3 days of adult life, as well as the visit duration over the 5 days of experimentation. We observed neither differences between male and female for all the proportions of visit durations (Poisson mixed-model, 3 degrees of freedom χ^2^ = 3.66, *p = 0.301*) (Figure 5E), nor for the variance of visit duration over the 5 days of adult life (Fisher-Snedecor’s test, F_9,9_ = 1.42, *p = 0.613*) (Figure 5F).

### 3.3 Photic environment controls feeding dynamics through clock-dependent and independent pathways

**FIGURE 6:**
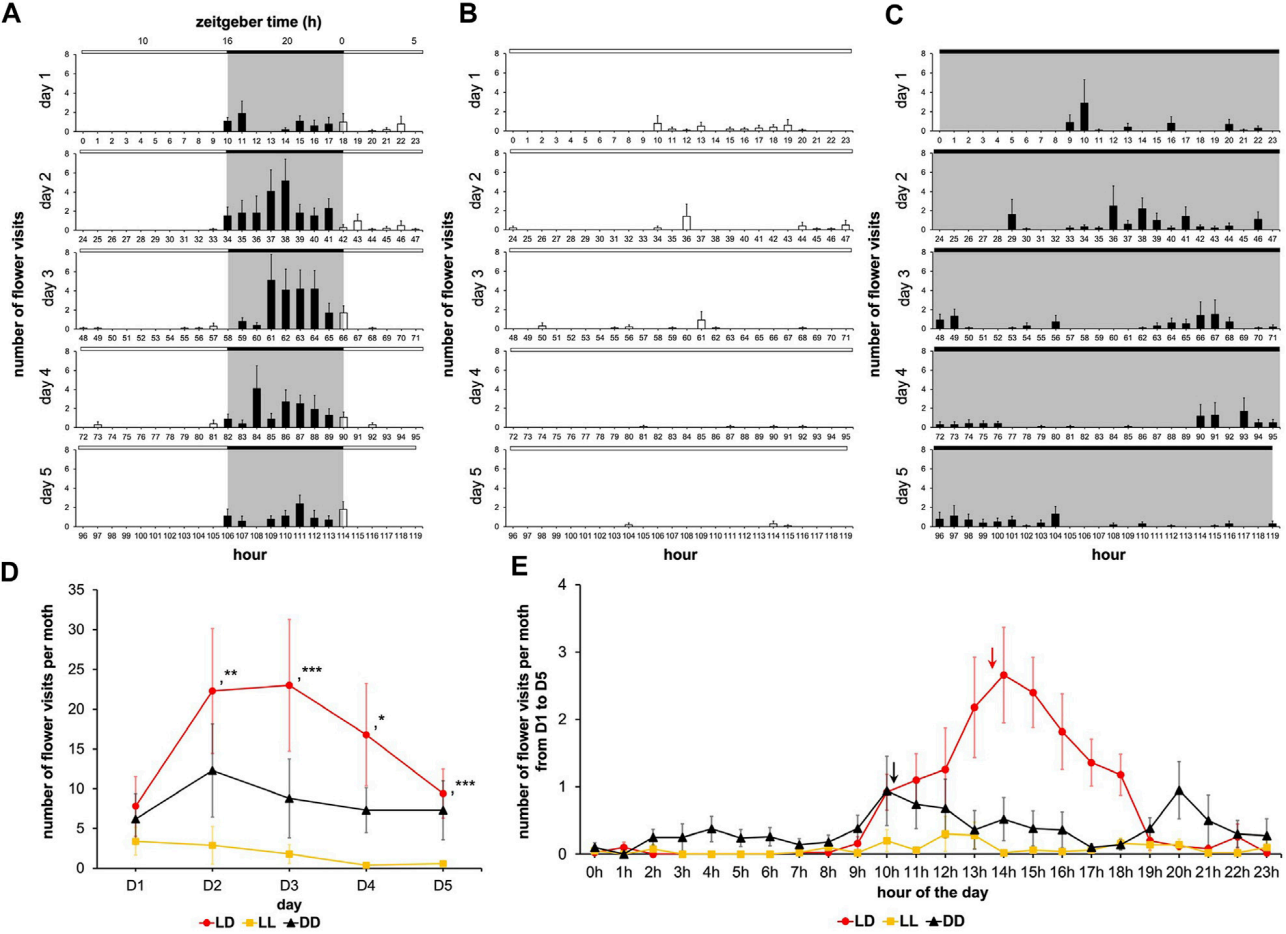
Feeding rhythms and feeding dynamics are gated by light. A first cohort was composed of males subjected to 16L:8D noted LD; a second cohort was composed of males subjected to 24L:0D noted LL; a third cohort was composed of males subjected to 0L:24D noted DD. Values correspond to the mean ± SEM (*n* = 10 for each cohort). Diagram for males subjected to 16L:8D **(A)** (same diagram as in Figure 2A), 24L:0D **(B)** and 0L:24D **(C)** (full bar, flower visit during scotophase; empty bar, flower visit during photophase). **(D)** Mean visit number per moth for the cohorts subjected to conditions LD, LL, and DD depending on the days (red, LD condition; black, DD condition; yellow, LL condition). Wilcoxon’s test (*, *p < 0.05*; **, *p < 0.01*; ***, *p < 0.001*) was performed to compare the cohorts, and statistical data are presented in Supplementary Table S5. **(E)** Mean visit number per moth for the cohorts subjected to conditions LD, LL, and DD according to the time of day; the raw data in Figure A, B and C acquired during the 5 days of experimentation were cumulated (red line, LD condition; red arrow, phase for LD condition; black line, DD condition; black arrow, phase for DD condition; yellow line, LL condition). Statistical analysis of the LD circadian rhythm was made are presented in Supplementary Table S6.

Light is the environmental parameter that undergoes the most pronounced change on a daily basis, therefore, we sought to determine the role of the photic environment on feeding dynamics. We investigated the effect of lighting conditions on adult males’ feeding dynamics by changing the daily photoperiod from 24-h to 16-h to 0-h, *i.e.*, LL, LD, and DD conditions respectively. The diagrams under LD, LL, and DD conditions highlight light-dependent modulation of the feeding rhythm (Figures 6A–C). The flower visitation frequency on the first day of adult life was not different across the LD, LL, and DD conditions (Wilcoxon’s test, W ≤ 62.00, *p ≥ 0.353*) (Figure 6D), since the cohorts underwent development under identical photic (LD) conditions. During the next 4 days of adult life, the frequency of flower visits did not significantly differ between LD and DD conditions (Wilcoxon’s test, W ≤ 74.50, *p ≥ 0.060*) but in LL, the number of the flower visits fell dramatically compared to LD (Wilcoxon’s test, W ≥ 80.50, *p ≤ 0.015*) (Figure 6D; Supplementary Table S5), while the individual visit duration became significantly more variable in LL (Fisher-Snedecor’s test, F_8,5_ ≥ 0.003, *p < 0.001*) (Figure 8A). Furthermore, rhythmic attributes of feeding behavior were greatly influenced by the ambient lighting conditions. Indeed, as soon as the moths were confronted with a monophotic environment from D1 to D5, the circadian rhythm of feeding behavior was disrupted or even completely disappeared (Figure 6E): the rhythm was distinguishable for the DD condition (Cosinor & ARSER models, *p < 0.001*) with a period equal to 50 h (*i.e.*, a 25 h multiple) (*p < 0.0001*), whereas the rhythm totally disappeared in LL condition (Cosinor & ARSER models, *p ≥ 0.772*).

**FIGURE 7:**
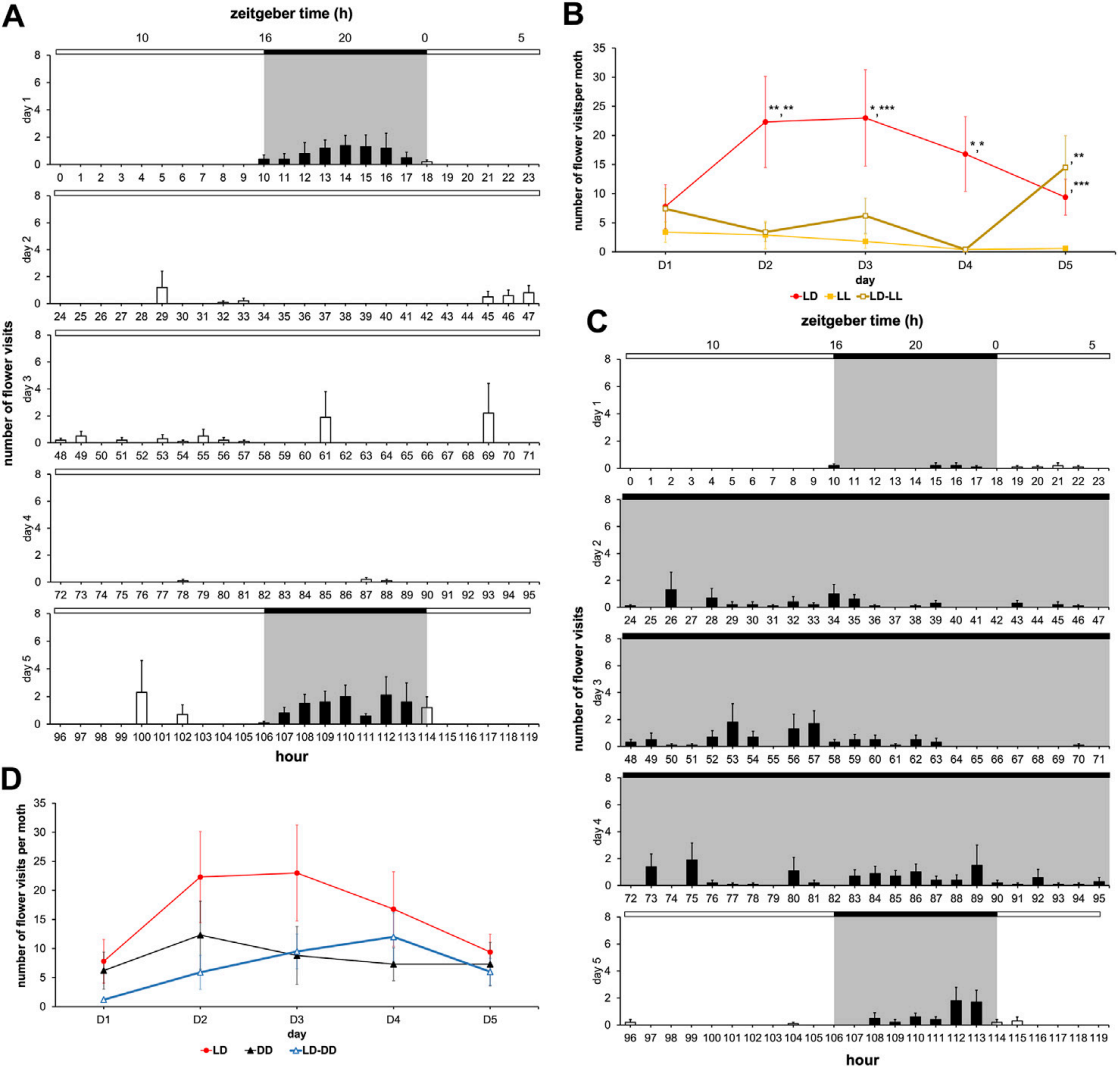
Interaction of the circadian clock with the acute effects of light patterns feeding behavior. Diagrams for males with a sugar solution available *ad libitum* and variation in number of visits and rhythm of feeding behavior according to photic environment. A first cohort was composed of males subjected to 16L:8D noted LD; a second cohort was composed of males subjected to 24L:0D noted LL; a third cohort was composed of males subjected to 0L:24D noted DD; a fourth cohort was composed of males subjected to 16L:8D on D1 and D5, and 24L:0D from D2 to D4 noted LD-LL; and a fifth cohort was composed of males subjected to 16L:8D on D1 and D5, and 0L:24D from D2 to D4 noted LD-DD. Values correspond to the mean ± SEM (n = 10 for each cohort). **(A)** Diagram for males subjected to 16L:8D on D1 and D5, and 24L:0D from D2 to D4 (full bar, flower visit during scotophase; empty bar, flower visit during photophase). **(B)** Mean visit number per moth for the cohorts subjected to conditions LD, LL, and LD-LL depending on the days (red, LD condition; black, DD condition; brown, LD-LL condition). Wilcoxon’s test was performed to compare the cohorts, and statistical data are presented in Supplementary Table S7. **(C)** Diagram for males subjected to 16L:8D on D1 and D5, and 0L:24D from D2 to D4 (full bar, flower visit during scotophase; empty bar, flower visit during photophase). **(D)** Mean visit number per moth for the cohorts subjected to conditions LD, DD, and LD-DD depending on the days (red, LD condition; black, DD condition; blue, LD-DD condition). Wilcoxon’s test was performed to compare the cohorts, and statistical data are presented in Supplementary Table S8.

The absence of rhythms in LL condition could have been brought about by a lack of training since the emerged adult’s circadian clock was immediately exposed to the constant light. On the other hand, the low flower visit frequency in LL condition could be a direct consequence of the moth’s inability to associate the artificial flower with sugar solution availability in this monophotic condition, since learning and memory are known to be regulated by lighting conditions in insects (Inami et al., 2020) and affect the content of the learning (Gonulkirmaz-Cancalar et al., 2023). To ascertain these, we subjected the male moths to the LD cycle on the first day of adult life, then left them in LL condition from D2 to D4, while on the last, *i.e.*, the fifth day of experimentation the LD cycle was reintroduced (Figure 7A). In the LD-LL regimen, the daily number of flower visits decreased from D2 to D4 (LL), and then significantly increased on D5 (LD) (Figure 7B; Supplementary Table S7). Flower visitation frequency during D2-D4 was lower than that of the monotonic LD condition (Wilcoxon’s test, W ≥ 80.00, *p ≤ 0.024*) and indistinguishable from the monotonic LL condition described earlier (Wilcoxon test, W ≤ 41.00, *p ≥ 0.451*) (Figures 6A, B). On D5 of the LD-LL regimen, the number of flower visits became equal to that of the LD condition (Wilcoxon’s test, W = 48.00, *p = 0.909*), and markedly differed from that of the LL condition (Wilcoxon’s test, W = 14.00, *p = 0.005*) (Figure 7B; Supplementary Table S7). Therefore, even a previously trained moth loses its rhythm under LL condition that degrades clock functioning and also reveals the suppressive effect of ambient light on the feeding behavior.

**FIGURE 8:**
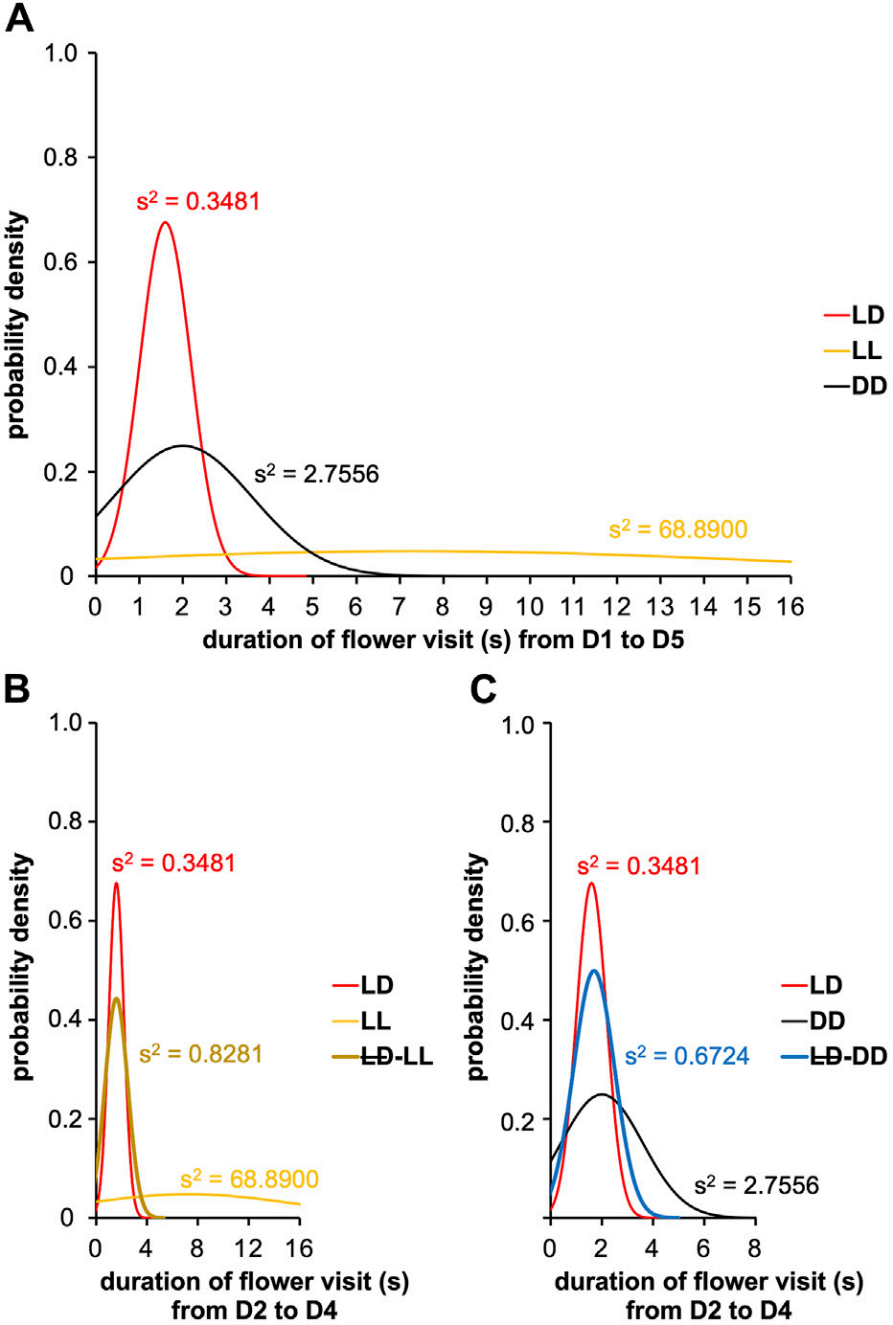
Visit duration of artificial flower according to ambient photic environment. The experiment was carried out on males with a sugar solution available *ad libitum* over 5 days with a variable photoperiod. A first cohort was composed of males subjected to 16L:8D noted LD; a second cohort was composed of males subjected to 24L:0D noted LL; a third cohort was composed of males subjected to 0L:24D noted LL; a fourth cohort was composed of males subjected to 16L:8D on D1 and D5, and 24L:0D from D2 to D4 noted LD-LL; and a fifth cohort was composed of males subjected to 16L:8D on D1 and D5, and 0L:24D from D2 to D4 noted LD-DD. Curves represent normal laws fitted to the data (s^2^ is the variance). **(A)** Duration of flower visit from D1 to D5 for the cohorts subjected to conditions 16L:8D (LD, *n* = 10), 24L:0D (LL, *n* = 6), and 0L:24D (DD, *n* = 9) (red, LD condition; black, DD condition; yellow, LL condition). A Fisher-Snedecor’s test was performed to analyze the variance of data. **(B)** Duration of flower visit from D2 to D4 for the cohorts subjected to conditions LD (*n* = 10), LL (*n* = 5), and LD-LL (*n* = 6) (red, LD condition; black, DD condition; brown, LD-LL condition). For all the conditions, a Fisher-Snedecor’s test was performed to analyze the variance of data. **(C)** Duration of flower visit from D2 to D4 for the cohorts subjected to conditions LD (*n* = 10), DD (*n* = 9), and LD-DD (*n* = 8) (red, LD condition; black, DD condition; blue, LD-DD condition). For all the conditions, a Fisher-Snedecor’s test was performed to analyze the variance of data.

Next, we measured the flower visit duration from D2 to D4 between LD, LL, and LD-LL conditions (Figure 8B). Interestingly, a clear difference was observed with LL (Fisher-Snedecor’s test, F_4,5_ = 781.48, *p < 0.0001*), indicating that photic exposure made visit duration more variable. Indeed, complementary experiments in which we used an LD-DD regimen (Figure 7C), revealed that moderately longer flower visit duration typical of DD (DD visit duration > LD visit duration, Fisher-Snedecor’s test, F_8,9_ = 7.67, *p < 0.001*) (Figure 8A) is brought down to the shorter values typical of LD, if and when the DD days (D2-D4) were preceded by a single day of LD (Fisher-Snedecor’s test, F_9,7_ = 2.13, *p = 0.332*) (Figure 8C). Notably, the feeding dynamics (Figure 7D) and the rhythms in flower visit frequency did not differ between monotonic DD and LD-DD conditions in terms of period and phase (Supplementary Table S9), suggesting that pre-imaginal training is sufficient to drive the adult circadian clock that regulates feeding behavior.

## 4 Discussion

*Agrotis ipsilon* is a nocturnal pest moth found all over the world, with the most important occurrence in the United States, France, Canada, and the UK (Rings et al., 1975; Global Biodiversity Information Facility, 2023). Such a global distribution is linked to its significant capacity to migrate seasonally to find new sources of food and optimal climactic conditions (Causse et al., 1989; Alarcón et al., 2008). Migration is very costly in terms of energy and requires a substantial food supply (O’Brien, 1999). In adult Lepidoptera, energy comes from nutrients taken from the nectar of flowering plants (Nepi et al., 2012; Broadhead and Raguso, 2021; for a review, see Roy et al., 2017). Although its natural repertoire of foraged flowers is not known, previous studies suggest that *A. ipsilon* feeds on sugar-rich nectars (Hostachy et al., 2019; Force et al., 2023). *A. ipsilon* moth has a short proboscis, the length of which is approximately half its body length. The morphology of its mouth parts seems suitable to feed on nectars from flowers with short corollas, as suggested by other works on moths (Krenn, 1990; 2010). A recent study (Force et al., 2023) also showed that the speed of sexual maturation in *A. ipsilon* adults is determined by the quality of its nectar diet. Therefore, *A. ipsilon* is a model of adult Lepidoptera whose feeding behavior is crucial for fitness. Understanding long-term feeding dynamics in this model, thus, becomes an important issue for behavioral ecology and at the same time opens the door toward applied research on pest control.

In our study, the artificial flower in the moth-box was adapted to the morphology of our insect model. Knowing that feeding is associated with global locomotor activity in moths (Broadhead et al., 2017; Niepoth et al., 2018; Wang et al., 2021; Zhang et al., 2021), we validated that we specifically measure the number and duration of flower visits and can display their dynamics as a function of time. Daily rhythms in these parameters were clearly visible in our results. The lower initial male performance could result from an initial lower motivation for food, probably due to larval-inherited food reserve in D1 moths. Alternatively, the number of flower entrances increased between D1 and D3 in males (but not females) without a significant increase in global locomotor activity suggests insects learned that entering the artificial flower yields food. In this case, this would be a case of operant conditioning with food as the reinforcer (for a review, see Staddon and Cerutti, 2003); such learning would only occur (or be expressed) in the scotophase, reflecting the nocturnal ecology of *A. ipsilon*. Thus, it would be related to the well-known abilities of bees to take into account day time in their learning (Gonulkirmaz-Cancalar et al., 2023). The gradual decrease in the number of flower visits induced by stopped availability of food (*i.e.,* removal of the reinforcer) would reflect extinction. Modifying the moth-box with two juxtaposed artificial flowers, with different shapes, and sugar solution content, could test whether learning actually occurs and would provide important insight into feeding cues, sensory discrimination, food preference, and their circadian dynamics.

While key behavioral drives like courtship and sleep are well known to be highly sexually dimorphic, very little has been known about the sex differences in feeding, in any insect model. Our results highlighted a greater impact of age on female moth’s feeding behavior. While the males visited the artificial flowers during the first 5 days of adult life with a similar frequency, in females, visit numbers started to drop after the third day. This could be related to the sex-specific state of reproductive maturation in *A. ipsilon*. Indeed, under standard laboratory feeding conditions, a developmental transition from a sexually immature to reproductively mature state occurs between the third and the fifth day of adult life in males (Gemeno and Haynes, 2000), while in females it takes place by the third day of adult life (Stanley et al., 1977; Gadenne, 1993). It is therefore likely that feeding behavior is linked to the state of sexual maturity of moths. We had shown that adding diversified sugars and sodium in the male diet accelerated sexual maturation to the third day of adult life (Force et al., 2023). Hence, we hypothesize such diets would affect flower visits in males.

An important sexual dimorphism in the peak timing of flower visits was underlined by our data. Females’ feeding behavior peaked earlier than males. We note that this phase difference in feeding behavior is coherent with the phase difference in courtship of females and males. Female *Agrotis segetum* has been shown to rhythmically produce sex pheromones, with maximal production in mid and late scotophase (Rosén, 2002; Závodská et al., 2009). A close relationship between sex pheromone production and pheromone release, *i.e.*, the calling activity of the female has been widely established in female moths (Mazor and Dunkelblum, 2005). Interestingly, the global locomotor activity of the female moth decreases during calling (Broadhead et al., 2017). Taken together, we speculate that *A. ipsilon* females visit flowers at the beginning of the scotophase before the onset of their sexual behavior. On the contrary, the period of the highest sensitivity to the sex pheromone in males, which is in the middle of the scotophase in *A. segetum*, is accompanied by an increase in global locomotor activity, coinciding with female calling behavior (Rosén et al., 2003). Given that feeding is associated with global locomotor activity in male moths (Wang et al., 2021), we propose that male *A. ipsilon* visits flowers mostly in the middle of the scotophase in nature.

Duration of the daily photophase has a strong impact on adult insects’ reproductive development, sexual behaviors, and locomotor rhythm (Delisle and McNeil, 1986; Dolezal et al., 2007). Thus, we investigated the effect of different lighting conditions on feeding behavior and its circadian variation in the males of *A. ipsilon*. In LL condition that stops the internal circadian clock of insects, feeding behavior became highly arrhythmic, while in DD the rhythm persisted weakly albeit with a slightly longer period, underscoring its free-running nature. In the presence of constant light (LL), we additionally observed a robust diminution of flower visitations during the five first days of adult life. Our results had remarkable similarity with the general conclusion of Xu *et al.* (2008) which showed clock-mediated as well as light-regulated daily rhythms of feeding in *Drosophila*. In insects, behavioral circadian rhythms are programmed by a central oscillator controlled by the cyclical expression of genes in the brain’s specific neurons. Indeed, the expression of the clock genes *period* (*per*) and *timeless* (*tim*), as well as *cryptochrome-2* (*cry2*) in certain insects such as moths and butterflies, is driven by the CLOCK (CLK)/CYCLE (CYC) heterodimer (Niepoth et al., 2018). In addition, *per* and *tim* gene expressions have been studied in several adult Lepidoptera including moths such as *Spodoptera frugiperda* and *Ephestia kuehniella* (Reppert et al., 1994; Froy et al., 2003; Iwai et al., 2006; Zhu et al., 2008; Kobelková et al., 2015; Hänniger et al., 2017). In these species, *per* and *tim* gene expressions oscillate over 24 h and are highest during scotophase, which is consistent with the moths’ circadian behaviors mainly observed during the night as in *A. ipsilon*. Furthermore, it is well known that environmental conditions such as temperature and photoperiod affect the amplitude and cycling of *per* and *tim* expressions (Pittendrigh et al., 1991; Iwai et al., 2006; Montelli et al., 2015). In nocturnal Lepidoptera, clock gene expression is attenuated during photophase (Iwai et al., 2006; Schuckel et al., 2007). It is therefore reasonable to think that this could also be the case in *A. ipsilon*, thus explaining the loss of the circadian rhythm of feeding behavior in LL condition. Injection of dsRNA targeting clock genes to the brain (*clk*, *cyc*, *per*, *tim* and *cry2*) could help us to identify the specific oscillator that drives feeding behavior rhythm by using the moth-box for instance. It will also be interesting to determine which photoreceptors–retinal rhodopsins, ocellar opsin, or deep-brain cryptochrome and/or opsin mediate the suppressive effect of light on feeding behavior in a non-model insect (for reviews see Hardin, 2011; Senthilan et al., 2019).

Moths subjected to constant daylight not only exhibited a highly reduced number of flower visits but also had a longer duration of these visitations. Such a change hints at the presence of a feeding homeostat. While the connection of the *pars intercerebralis* with the neurohemal organs has been known as the central axis for hunger and satiety regulation (for a review, see Franco et al., 2018), surprisingly little is known about the neuroendocrine control of the time-dependency in the microarchitecture of feeding behavior. Fat body could be a crucial player in the regulation of this behavior. Indeed, studies show that the clock present in the fat body regulates feeding behavior and suggest that the fat body and neural clocks are coordinated in opposite ways to provide an optimal metabolic state (Xu et al., 2008; for a review, see Franco et al., 2018). Moreover, the fat body clock regulates the expression of many of its transcripts involved in metabolism and reproduction; among these cyclic genes, some are controlled by external factors such as light (Xu et al., 2011). The robustness of our new experimental device would be a great help to unravel this. Finally, our quantitative analyses of feeding behavior in the nocturnal crop pest moth *A. ipsilon* paves the way to tackle the seemingly complex relationship between feeding, mating, and sleep-wake rhythms. It will also allow scientists to address how linear developmental time and cyclic circadian time intersect and influence each other. From the perspective of agricultural pest management, our device could also be harnessed to screen for natural feeding suppressants that could arrest or the delay reproductive maturation of pest moth species.

## Data Availability

The raw data supporting the conclusion of this article will be made available by the authors, without undue reservation.

## Supplementary material

SUPPLEMENTARY FIGURE S1 Distribution of flower visit duration in male moths with a sugar solution available from D1 to D5 and submitted to photoperiod 16L:8D (*n* = 10). The analysis was made on the second and the third day of experimentation according to three-time slots of scotophase (10 h–12 h, 13 h–15 h, and 16 h–18 h).
