# Supplementary material for: Regulation of feeding dynamics by the circadian clock, light and sex in an adult nocturnal insect
Source: Front Physiol. 2024 Jan 9;14:1304626. doi: 10.3389/fphys.2023.1304626 (PMC10803417; doi:10.3389/fphys.2023.1304626)
Supplement: Supplementary file 2 [file Table8.DOCX]

**Supplementary Table S8.** Detailed analysis of data from Figure 7D (n = 10 for each cohort).

|  | | **Days** | | | | |
| --- | --- | --- | --- | --- | --- | --- |
|  |  | **D1** | **D2** | **D3** | **D4** | **D5** |
| **Pairwise comparisons**  Wilcoxon’s exact test p-value | LD vs. DD | 0.877 | 0.080 | 0.060 | 0.490 | 0.195 |
|  | LD vs. LD-DD | 0.351 | 0.058 | 0.121 | 0.671 | 0.342 |
|  | DD vs. LD-DD | 0.371 | 0.686 | 0.374 | 0.562 | 0.907 |
