# Supplementary material for: Regulation of feeding dynamics by the circadian clock, light and sex in an adult nocturnal insect
Source: Front Physiol. 2024 Jan 9;14:1304626. doi: 10.3389/fphys.2023.1304626 (PMC10803417; doi:10.3389/fphys.2023.1304626)
Supplement: Supplementary file 4 [file Table7.DOCX]

**Supplementary Table S7.** Detailed analysis of data from Figure 7B (n = 10 for each cohort).

|  | | **Days** | | | | |
| --- | --- | --- | --- | --- | --- | --- |
|  |  | **D1** | **D2** | **D3** | **D4** | **D5** |
| **Pairwise comparisons**  Wilcoxon’s exact test p-value | LD vs. LL | 0.353 | 0.002 | < 0.001 | 0.015 | < 0.001 |
|  | LD vs. LD-LL | 0.908 | 0.003 | 0.024 | 0.015 | 0.909 |
|  | LL vs. LD-LL | 0.209 | 0.562 | 0.451 | 1 | 0.005 |
