# Supplementary material for: Regulation of feeding dynamics by the circadian clock, light and sex in an adult nocturnal insect
Source: Front Physiol. 2024 Jan 9;14:1304626. doi: 10.3389/fphys.2023.1304626 (PMC10803417; doi:10.3389/fphys.2023.1304626)
Supplement: Supplementary file 5 [file Table9.DOCX]

**Supplementary Table S9.** Detailed analysis of the rhythms in feeding frequency between standard and monotonic LD-DD conditions. A treatment-dependent dampening adjustment was not significant and was therefore dropped. The reference group was LD; values for the LD-DD group are the sum of the reference group coefficient and the coefficient of the LD-DD group. Values correspond to the coefficient ± SE (n = 10 for each cohort).

|  | **Value** | **p-value** |
| --- | --- | --- |
| γ (dampening) | 0.0017 ± 0.0015 | 0.248 |
| A | 0.4052 ± 0.0912 | < 0.0001 |
| φ (phase) for LD | 13.6973 ± 0.2602 | < 0.0001 |
| φ for LD-DD | 15.4516  (coefficient = 1.7543 ± 3.7137) | 0.637 |
| τ (period) for LD | 24.1539 ± 0.1024 | < 0.0001 |
| τ for LD-DD | 49.0019  (coefficient = 24.8480 ± 3.1940) | < 0.0001 |
| B, a, and b are random factors. |  |  |
