# Supplementary material for: Regulation of feeding dynamics by the circadian clock, light and sex in an adult nocturnal insect
Source: Front Physiol. 2024 Jan 9;14:1304626. doi: 10.3389/fphys.2023.1304626 (PMC10803417; doi:10.3389/fphys.2023.1304626)
Supplement: Supplementary file 6 [file Table2.DOCX]

**Supplementary Table S2.** Detailed analysis of data from Figure 4A. A treatment-dependent dampening adjustment for D1 and D2 was not significant and was therefore dropped. The reference group was males with a sugar solution available from D1 to D5; value for the males with a sugar solution available on D1 and D2 group is the sum of the reference group coefficient and the coefficient of the second group. Values correspond to the coefficient ± SE (n = 10 for each cohort).

|  | **Value** | **p-value** | |  |
| --- | --- | --- | --- | --- |
| γ (dampening) | -0.0025 ± 0.0024 | 0.307 | |  |
| A | 0.4260 ± 0.1087 | < 0.0001 | |  |
| φ (phase) for male with a sugar solution available from D1 to D5 | 13.7267 ± 0.2571 | < 0.0001 | |  |
| φ for male with a sugar solution available on D1 and D2 | 43.4482  (coefficient = 29.7215 ± 1.3800) | < 0.0001 | |  |
| τ (period) for male with a sugar solution available from D1 to D5 | 24.1431 ± 0.1035 | < 0.0001 | |  |
| τ for male with a sugar solution available on D1 and D2 | 42.3383  (coefficient = 18.1962 ± 1.5542) | < 0.0001 | |  |
| B, a, and b are random factors. |  | |  | |
