# Supplementary material for: Regulation of feeding dynamics by the circadian clock, light and sex in an adult nocturnal insect
Source: Front Physiol. 2024 Jan 9;14:1304626. doi: 10.3389/fphys.2023.1304626 (PMC10803417; doi:10.3389/fphys.2023.1304626)
Supplement: Supplementary file 7 [file Table5.DOCX]

**Supplementary Table S5.** Detailed analysis of data from Figure 6D (n = 10 for each cohort).

|  | | **Days** | | | | |
| --- | --- | --- | --- | --- | --- | --- |
|  |  | **D1** | **D2** | **D3** | **D4** | **D5** |
| **Pairwise comparisons**  Wilcoxon’s exact test p-value | LD vs. LL | 0.353 | 0.002 | < 0.001 | 0.015 | < 0.001 |
|  | LD vs. DD | 0.877 | 0.080 | 0.060 | 0.490 | 0.195 |
|  | LL vs. DD | 0.361 | 0.301 | 0.249 | 0.023 | 0.090 |
