# Supplementary material for: Regulation of feeding dynamics by the circadian clock, light and sex in an adult nocturnal insect
Source: Front Physiol. 2024 Jan 9;14:1304626. doi: 10.3389/fphys.2023.1304626 (PMC10803417; doi:10.3389/fphys.2023.1304626)
Supplement: Supplementary file 8 [file Table3.DOCX]

**Supplementary Table S3.** Detailed analysis of data from Figure 5C (n = 10).

|  | | **Days** | | | | |
| --- | --- | --- | --- | --- | --- | --- |
|  |  | **D1** | **D2** | **D3** | **D4** | **D5** |
| **Global test** | Wilcoxon’s exact test p-value | 0.145 | 1 | 0.150 | 0.168 | 0.012 |
