# Supplementary material for: Regulation of feeding dynamics by the circadian clock, light and sex in an adult nocturnal insect
Source: Front Physiol. 2024 Jan 9;14:1304626. doi: 10.3389/fphys.2023.1304626 (PMC10803417; doi:10.3389/fphys.2023.1304626)
Supplement: Supplementary file 9 [file Table4.DOCX]

**Supplementary Table S4.** Detailed analysis of data from Figure 5D. A treatment-dependent dampening adjustment was not significant and was therefore dropped. The reference group was male; value for the female group is the sum of the reference group coefficient and the coefficient of the female group. Values correspond to the coefficient ± SE (n = 10 for each cohort).

|  | **Value** | **p-value** |
| --- | --- | --- |
| γ (dampening) | -0.0008 ± 0.0025 | 0.759 |
| A | 0.4330 ± 0.1292 | < 0.001 |
| φ (phase) for male | 13.6956 ± 0.2807 | < 0.0001 |
| φ for female | 35.9178  (coefficient = 22.2222 ± 2.4730) | < 0.0001 |
| τ (period) for male | 24.1524 ± 0.1120 | < 0.0001 |
| τ for female | 48.9089  (coefficient = 24.7565 ± 2.7828) | < 0.0001 |
| B, a, and b are random factors. |  |  |
