# Supplementary material for: Regulation of feeding dynamics by the circadian clock, light and sex in an adult nocturnal insect
Source: Front Physiol. 2024 Jan 9;14:1304626. doi: 10.3389/fphys.2023.1304626 (PMC10803417; doi:10.3389/fphys.2023.1304626)
Supplement: Supplementary file 10 [file Table6.DOCX]

**Supplementary Table S6.** Detailed analysis of data from Figure 6E. Sex-dependent adjustments were not significant and were therefore dropped. Values correspond to the coefficient ± SE (n = 10 for each cohort).

|  | **Value** | **p-value** |
| --- | --- | --- |
| γ (dampening) | -0.0022 ± 0.0021 | 0.284 |
| A | 0.3638 ± 0.0930 | < 0.001 |
| φ (phase) for LD | 13.7241 ± 0.2478 | < 0.0001 |
| φ for DD | 47.9004  (coefficient = 34.1763 ± 0.9681) | < 0.0001 |
| τ (period) for LD | 24.1433 ± 0.0996 | < 0.0001 |
| τ for DD | 50.1778  (coefficient = 32.6707 ± 6.4711) | < 0.0001 |
| B, a, and b are random factors. |  |  |
